# Dr. Yeats's Explanation to Mr. Gibbon

**Published:** 1813-12

**Authors:** G. D. Yeats

**Affiliations:** Bedford


*To the Editor of the Medical and Physical Journal.*

SIR,

INSTEAD of entering into a discussion of a medical question, I find myself obliged to controvert a charge of unfairness attributed to me, with what justice let those judge who take the trouble of reading this correspondence. I must repeat by positive assertion that I never understood the original difference of opinion, whatever may have subsequently occurred, to have taken place from any other cause than solely from the symptom of vomiting of urine. I then entirely understood that as that was an impossible occurrence, the girl must have practised imposition. Here most probably the difference of opinion would have rested for ever without any further discussion; but what were the steps taken in consequence of this difference of opinion? A meeting of medical gentlemen is held at the request of Mr. Gibbon, all of whom had never met before, without any previous knowledge on my part that such meeting was to be held, and when I was from home. I understand the meeting to have been a casual one; but still it should have been postponed, as I could not be present, especially too as the meeting was for the express purpose of investigating a subject in which, as Mr. Gibbon himself states, I was very much concerned. Moreover, the impropriety of holding such a meeting in my absence was hinted to him. If all this be accidental, it nevertheless wears an ungracious appearance. A paper is drawn up at this meeting declaratory of the

 opinion opinion that the vomiting of urine, as stated, appeared to be utterly impossible. Here again the difference of opinion between Mr. Gibbon and myself would have probably rested for ever; but what is next done? This document is submitted to the perusal of respectable persons, who say to me, "Well, doctor, you are quite wrong; vomiting of urine is utterly impossible!" In what predicament did I then stand? I must either have remained tranquil under the charge of having asserted the probability of the occurrence of a physiological fact which was understood to be declared by the profession to be an utter impossibility, or to have taken up the pen in my own defence: no man could hesitate which alternative to adopt.

This was exactly the cause which gave rise to the paper on Ischuria; and the form, manner, and nature of the publication, were calculated completely for medical discussion, without reference to any previous difference of opinion; thus endeavoring to avoid a protracted dispute about words misunderstood, expressions forgotten, and meanings misapplied. All I had in view was, to show that urinous vomiting was not so impossible an occurrence as some imagine, and that I was not singular in holding that opinion.

I may be allowed this explanation of my own feelings of what I originally understood. I unequivocally disclaim any intention of either directly or indirectly reflecting upon any individual whatsoever. If, therefore, Mr. Gibbon feels hurt, I cannot see why: he must thank himself for having obliged me to produce that paper in my own defence, and to impugn opinions circulated against mine. I have avoided discussing the nature of imposture, as that must depend upon a judicial inquiry into the credibility of human testimony, never having personally witnessed the disputed symptom. I am, however, very unwilling to condemn and consign to infamy any one from the statement of an uncommon occurrence, on the ground of the assertion of the impossibility of it, when I have proof that such an occurrence has taken place in various persons under nearly similar circumstances.

That Mr. Gibbon is satisfied his reasons are sufficient for his conviction against the story of the girl, I do not doubt; but surely he should allow me the privilege of reasons for my conviction also. I can have no objection to give a statement of the circumstances of the case; but whatever intention I may have had so to do, it is now a good deal repressed by the manner in which Mr. Gibbon has published his narrative. Personal reflections will not contribute to elucidate the subject. I could never hesitate to acknowledge the detection of an imposture, when clearly detected.

 Much

Much wiser heads than mine have been deceived by the artful contrivances of mankind; but I cannot agree that a person is an impostor, because professional men are divided in their opinions on the question of urinous vomiting.

As connected with this subject, I shall be much obliged, sir, by an insertion in your next Number of the accompanying case of vomiting of urine, extracted from the Philadelphia Transactions, a book in comparatively very few hands in this country.* I wish it to be inserted as a case of reference, and of much curiosity on this particular subject; and as further showing that urinous vomiting is not quite so fabulous as Mr. Gibbon thinks.

I must now advert to some other matters in Mr. Gibbon's paper, which, instead of being a dignified and dispassionate investigation, a calm statement of the reasons on which his conviction is founded, carries with it all the appearance of an angry *tirade* of personal reflection, and containing circumstances, from misunderstanding no doubt, which it is incumbent on me to correct.

I first saw Ann Foulkes about May 1808, by the desire of a very respectable dissenting minister, from his commiseration of the great sufferings under which she labored. My humble exertions to relieve were ineffectual. I was obliged, therefore, to leave her to her fate; and I saw and heard but very little of her till the winter of 1811. During this interval the druggist visited this girl of his own accord, from motives of humanity, and prescribed according to his own judgment unconnected with me. When I saw her in 1811, it was at a period long after the commencement of the vomiting of urine; I therefore could have nothing to do in the sanction or control of the original exhibition of the remedies, whatever opinion I may have given of their application. A list of medicines which had been given was shown to me. The druggist or any person would be of equal use, for no one could render her any service, was an observation which I recollect to have expressed. The tumefaction in the epigastric region, at all the times that I ever saw it, was in my estimation a distension of the stomach; and so is the opinion o other medical gentlemen besides myself who

* When the circumstances and doubts of this girl's story were bruited abroad, many persons of curiosity and observation were desirous of knowing how far any analogous histories were upon record. This book, as containing Dr. Senter's case of urinous vomiting, was therefore lent by me to them. This is one among other reasons why I wish the case extracted from it to be published, for others who have not seen it, and who have applied to me for similar information.

examined examined it. It was the complaint to all appearance like that to which Sauvages gives the name of Meteorismus Ventriculi, a complaint of no very uncommon occurrence in certain morbid conditions of the stomach, with which this girl has been long afflicted.

I know not how to reconcile the account given by Mr. Gibbon of her general good health, with the great suffering which I frequently witnessed. If she possesses the power, and practises the art of pursing together (if this be Mr. Gibbon's meaning, for the description is confused) the abdominal muscles, with a view to feign disease, no one could hesitate to pronounce her an impostor. It appears to me too complicated a contrivance for an ignorant country girl. But has Mr. Gibbon not been deceived by the convulsed state into which different parts of her frame have been frequently thrown, by morbid sympathy, with a highly diseased stomach, arising from organic mischief? Has he not frequently witnessed the agitated state of the abdominal muscles, when examining that part of the body for disease? This occasional swelling and subsidence of the stomach was a symptom of her complaint many years before the vomiting of urine. With respect to the quantity of clothing, provision, and money sent to her, I know nothing; but animal food in a solid form gives great pain when it has been taken: she therefore cannot eat it. That almost any person may practise deceit and imposition, is an axiom not to be denied. What embellishments and exaggerations of the girl's situation may have been added by those around her, I cannot say.

But I will not lose sight of the question of urinous vomiting, the original matter of discussion, by which my whole attention has been excited, and which I think was by no means an impossible occurrence, connected with the girl's other symptoms, if the authenticated records and testimony of the great and good in our profession are to be relied on. The resistance to the use of the catheter, as stated by Mr. Gibbon, forms no ground for considering the girl an impostor. I myself never past it, which I now regret, for the satisfaction of others; but I believe that urinous vomiting may take place when no urine shall be found in the bladder, in the Ischuria Ureterica; and some professional men of eminence are of opinion that other glands besides the kidneys may put on an action so as to secrete urine. I do not say that I agree to this doctrine. I did not see the girl nor know of it till some time after the vomiting of urine had commenced; very few times after that to the period of Mr. Gibbon's visits; and after these, not once, I believe, till after the urine had returned to its natural channel, previous to which event I was not informed, to the best of my recollection, of the suspicion of imposition. It would have been well if Mr. Gibbon had informed me of the resistance to the use of the catheter. Time enough was allowed, for Mr. Gibbon visited her March 19th, and the urinous vomiting did not cease till on or about April 12th following.

During the whole of the period from the time that I was acquainted with the occurrence of the disputed symptom, I was much harassed with professional business, under considerable indisposition, though able to go abroad. I was also obliged to be in London, on private concerns, which occupied no inconsiderable portion of my time. Had moments of reflection allowed my mind to be impressed with the importance which the case has since assumed, or had even the slightest idea come across me of the practice of imposition, no circumstance of attention, however painful, or of scrutiny, however laborious, should have been omitted by me, even situated as I then was. I can only repeat what I have said in a former communication, that I credited the relation of those circumstances which I did not personally witness, from the extreme severity of affliction and suffering which very many most respectable persons, as well as myself, had previously, and have since frequently witnessed. I believed that this occurrence had happened to her as it happened to others, for I do not know how she could have taken it into her head to have invented it. It is matter of opinion,—I can say no more.

If Mr. Gibbon has incurred the ill opinion of persons whose favor he is solicitous to conciliate, it is not my fault; if he has involved himself in a dilemma by the mode of conducting the inquiry, I cannot help it. Instead of combating, by physiological discussion, the facts adduced in the paper on Ischuria, does Mr. Gibbon really think that the anonymous history of the tadpole Essex girl will have any weight with people of reflection, against the substantiated facts and opinions from the authority of such men as Haller, Morgagni, Sauvages, Heberden, and Gregory, whose works form the principal part of the principal shelf of the libraries of professional men? Is Mr. Gibbon really of opinion that this story can weigh against the sound judgment, the critical acumen, and the penetrating observation, with which these pillars of our profession have been universally acknowledged to be gifted?

How it can be involved I know not; I must nevertheless make my acknowledgments to Mr. Gibbon for his tenderness of my professional character. As the expression of it, however, ever, comes rather in a questionable shape, I may be allowed to say,

> Non tali auxilio nec defensoribus istis,
> Tempus eget.

The text is in Virgil; Mr. Gibbon will probably know where to look for the commentary.

I am, Sir,
Your very obedient Servant,
G. D. YEATS.

*Bedford,*

*Nov.* 9, 1813.

*An Account of a singular Case of Ischuria in a young Woman, which continued for more than three years; during which time, if her Urine was not drawn off with the Catheter, she frequently voided it by Vomiting, and for the last twenty Months, passed much Gravel by the Catheter, as well as by Vomiting, when the use of that Instrument was omitted, or unsuccessfully applied.* By ISAAC SENTER, M.D.

LUCY FOSTER, aged 15 years, a fleshy, healthy-looking, well-proportioned young woman, was taken, June 1st, 1785, with a pain in the left hypochondrium, accompanied with cough, fever, oppression at the chest, and difficulty of breathing. Being in very poor circumstances, her friends neglected asking advice till about a fortnight from her seizure, when I was called to her assistance. I was informed by her mother that she became a woman at thirteen, and continued pretty regular in her menses till within five weeks of her present illness; and that from her seeing nothing during that period, she supposed her to have taken a bad cold, as she was very inattentive to her health, and had been obliged to do the duty of a servant maid. Her pulse was upwards of 100 in a minute, her tongue coated with that sort of fur which often accompanies a bad kind of chronic inflammation of the thoracic viscera. I took ten ounces of blood from her arm, gave her an emetic, and directed a blister to the affected side. The blood, when cool, heaved up its coagulable lymph, as is common in pneumonic inflammation; but the buff was tender, and serum did not separate, as is usual in cases of acute inflammation of the breast. Expectorant febrifuge mixtures, &c. were given her, and another blister applied to her side within a few days. These medicines produced an abatement of the symptoms, and in the course of three weeks I ceased to visit her. I, however, looked upon her disease to have a strong tendency to a consumption; and about the fourth week from my first visiting her, she vomited up a quantity of bloody pus of a very disagreeable kind, which, with the preceding symptoms, symptoms, induced me to think a vomica had burst in her stomach; for during the whole of this illness, from my first seeing her, her stomach was so irritable that it was with difficulty food or medicine could be made to sit upon it; and she often vomited up the most simple barley drink. She had a suppression of urine for twenty-four hours, but did not get any aid from medicine, as nature relieved herself. She, however, became regular in her menses, and recovered so far in about two months as to return to her usual labor; and continued capable of doing her duty to the satisfaction of her employers till June following, 1786.

On the 3d of this month I was desired to visit her again, when I found all her old complaints (except the suppression of her menses) returned with greater severity than they appeared last year. She was now let blood, and treated in other respects as before, her distress continuing so great that I found it necessary to repeat the operation (drawing small quantities) several times, as nothing else appeared to afford her any considerable relief. Her tongue was covered with a yellowish coat in the middle, and a muslin color at the edges. Her pulse beat 120 strokes in a minute. The irritability of her stomach was so great, that it had become extremely difficult to give any article either of medicine or nourishment but what she vomited up immediately. The effervescent draughts, infusions of Columbo with spirits of sweet nitre and sweet vitriol, liq. anod. min. &c. were tried without any lasting effect. Opium gave the most permanent relief, and afforded her that refreshment from sleep which she could obtain by no other means. As I now looked upon her case to be of long continuance, and residing in a distant part of the town, I called but seldom, after the severity of her symptoms had subsided, which they did in about three weeks.

On the 2d of July she was seized with a total suppression of urine, without any perceptible cause, which continued five days, not being able to void a single drop; and notwithstanding her pain and distress were very great, she did not let her circumstances be fully known to her friends, for fear of having it drawn off with an instrument. The beginning of the sixth day she was taken with a vomiting, which lasted till she brought up nothing but water, which, she said, tasted in every respect like urine. As her vomiting continued, she found relief in the bottom of her belly from the swelling and great soreness she had felt for several days. She now thought herself much better, but her vomiting recurred the next day, as I was informed, and continued more or less every day till I saw her, which was on the 14th of the month.

As she had discharged from her stomach every thing she ate or drank from the time of her first vomiting till this, she did not suffer so much from the ischury which still continued, as she did before the first evacuation. I prevailed upon her to let me pass the catheter into the bladder, whence I drew about three pints of urine, clear, but high-colored. Her strength was very much exhausted, and she felt great heat and soreness throughout the abdominal viscera. A variety of medicines were prescribed, and every method pursued that could be thought of, to allay the extreme irritability of her stomach, and restore the natural action of her bladder.

For ten weeks successively she was incapable of retaining on her stomach either food or medicines, except opium: this was her only solace by day as well as by night. From this time to December she continued with very little abatement of her distress, or alteration of her circumstances; and, as she could lie in no other position, she was constantly supported in an arm chair, in a reclined posture, with pillows under her hips. Whenever I omitted to draw off her water once in thirty or thirty-six hours at farthest, she never failed to vomit it up. *To ascertain so extraordinary a fact beyond the possibility of a mistake on my part, or a deception on hers, I often visited her about the time I knew she must vomit, if the catheter was not introduced; and I examined her bladder, found it full, hard, and tender, and sat by her till the vomiting recurred, saved the water that she brought up this way, compared it with what I drew off, and found it the same in every respect.*

During the time her urine came off by vomiting, she suffered extreme anxiety, and always complained of great heat, smarting, and extreme thirst, and a sensation of inversion or turning up of something, (running, as she expressed it,) that appeared to tear her bowels. As the affair had become so lengthy, and my business was such that it was not in my power to attend upon her as often as her case required, I instructed the young gentlemen who lived with me in the use of the catheter, and they waited on her in my absence as often as they could conveniently.

In the month of January, 1787, from some cause unknown, she could not be relieved by the instrument, nor could she vomit up her urine for several days, when it passed off by the navel for three days successively; after which the catheter was used with the same effect as before. From this time to August following, there was so great a sameness in her complaints, that nothing occurred worth noticing. About the beginning of this month, a brick-colored gravel began to pass off through the catheter, and soon became so large and and plentiful, that neither urine or gravel could be completely evacuated by the instrument in its usual form. I had one made of a different construction, open at two of the sides for about half an inch, which answered my wishes. She continued to discharge gravel this way whenever her urine was drawn off, till the beginning of November, at which time she felt more distress than usual, whenever her urine came off by vomiting, and she soon observed a gritty substance in her mouth. When I was informed of this new phenomenon, I requested her to save the urine for my inspection the next time she vomited. I compared this with what I drew off, and found it contained the same kind of gravel as that which past the catheter. I procured and saved several drachms of this gravel that came from her both by the instrument and by vomiting, and could observe no difference in the color or consistence of them.

From this period to the summer 1788, her complaints continued much the same. When her water was not drawn off, she always brought it up by vomiting, commonly attended with great pain in the head. During this summer, she twice past a small quantity of urine through the urethra, in consequence of being frightened, once by thunder, and the second time by the falling of a window in her room. This served only to raise her spirits for a few days, with the expectation of her urine returning through its natural channel. Her case, however, continued the same in that respect, and grew every day more complicated in others. The hypogastrium became more tumid and tender, and her bladder appeared very much thickened and extremely sore even after it was evacuated ; add to this, the apparent inequality of the surface of the bladder was so great, and the tumor shifting sometimes towards the right and at others to the left inguen, according as her body was moved, that I began strongly to suspect a stone.

Through the month of September, her urine could very rarely be drawn off; for, upon the introduction of the catheter, a spasm seized the urethra and neck of the bladder; and, though the instrument appeared to pass high up into the fundus of the bladder, not more than a gill could be drawn before it stopped entirely, with a sensation of something falling down against its cervix, which she was very confident was a stone. In the course of this month she vomited more sand than she had at any time before, and failed in strength and spirits so fast, that I was apprehensive she would not live the month out. Her urethra, bladder, and external genital parts, were so extremely sore, that, for some time, it prevented prevented my searching her for the stone in the manner I intended.

About the beginning of October, I was able to introduce the sound, when I readily met with a stone which appeared of a small size, and rather softer than urinary calculi commonly are. I repeated the examination a number of times, till I was perfectly satisfied that this was the case. She would readily have undergone the operation of lithotomy, but I told her no lasting advantage could be expected from it while her viscera continued in such a diseased state. During this month her urine could be drawn off but part of the time, and *she vomited it up for more than a week, without the possibility of any relief from the instrument, notwithstanding it was kept in the bladder sometimes during the whole night.* She had, at different seasons of the year, several ill-conditioned small abscesses on her arm-pits, and on other parts of her body, but they did not appear to benefit her general complaints. She also voided at different times, by vomiting, (after she had thrown up all her urine,) a bloody pus, of a very disagreeable appearance and coppery taste. As her case was so very uncommon, I at different periods of it requested the advice of most of the faculty of this town. She was visited by the late Dr. Fletcher, Drs. Olyphant and Mason; the last of these gentlemen frequently attended her, both with me and in my absence, repeatedly relieved her by the catheter, *and saw her vomit up both urine and gravel.* She was also visited transiently by Dr. Waterhouse of Cambridge, and several other physicians of eminence, who belonged out of the State.

During the remainder of the fall, and principal part of the winter ensuing, the same troublesome sensation of the falling down of a stone in the bladder upon the use of the catheter continued, and induced the most excruciating pain and misery imaginable. She was put into different positions when the catheter was introduced, and I gave the instrument various directions in the bladder, sometimes with success, at others without. Her bowels for the most part were much less constipated than could have been expected, considering the frequency of vomiting, her supine situation, and the little nourishment she was able to retain upon her stomach; and during the whole of her disease, till within three months of her death, the catamenia were irregular. Sometimes they appeared every fortnight, and at others she past the regular period for that evacuation two or three months without having any; but it did not appear to me that her disease was much influenced by either. She had by times a dry cough, with the return of the old pain in the side; but she never expectorated by coughing any kind of purulent matter that could induce me to suppose her lungs were considerably diseased. The bloody matter that she brought up always came away by vomiting, preceded by a more morbid than ordinary irritability of the stomach, soreness, and extreme anxiety.

Early in the spring 1789, her urine began to pass per anum, loaded with the same kind of gravel that had come away by the catheter. This gave her some respite with respect to her vomiting, though she continued to throw up more or less urine as well as gravel that very week. This new course of her water gave her a very troublesome tenesmus; but the stone in the bladder, as well as the pain and disagreeableness arising from the sensation of its descent, became daily less fatiguing. Her strength and spirits decayed fast, and the fever that she had before continually labored under, grew more completely hectical. After the 13th of May, her bladder never became so much distended with urine as it had been before, and both this and the gravel now generally past her once in twenty-four hours, either by vomiting or purging. She however introduced the catheter herself, and sometimes drew off her urine to the quantity of a gill. The secretion of urine, as well as the formation of calculi, evidently diminished in proportion to her loss of strength, and the increase of the diarrhœa. The menses entirely ceased. During the latter part of spring and summer, she became quite paralytic at times; the frequency of vomiting increased, and she had several convulsion fits after vomiting. She grew more and more emaciated; her convulsions returned more frequently; her fever was more putrid; she at last became lethargic; and on the 11th of August, death, which she had long and ardently wished for, put a period to a series of the most complicated and singular misery that I have ever seen since my acquaintance with disease.

*Appearances on Dissection.*—Thorax.—In this cavity there was nothing appeared unnatural, except a considerable adhesion of the right lobe of the lungs to the pleura.

Abdomen.—The omentum was principally wasted, but not more than is commonly the case with those who die tabid. It was however of a dark gangrenous color pretty generally. The stomach appeared very much changed from its natural color, and in a gangrenous state, containing a semi-purulent matter, of a fœtid scent.

Liver and gall-bladder.—There were no preternatural adhesions of the former, nor gall-stones in the latter, and their color, &c. not unusual.

Intestines.—

Intestines.—In these there are no ruptures, either of their muscular coats, blood-vessels, or lymphatics, that we could discover. The villous coat was much destroyed, and the color of the intestines darker than is common, except the duodenum, which was very much discolored with the bile.

Kidneys and ureters.—In these there was no considerable deviation from a state of soundness; they were lax or flabby, but no rupture of any of their vessels, or any calculi discoverable.

Urinary bladder.—This was in its natural situation, not the least thickened, had no sand or gravel in it, nor did it adhere preternaturally to any of the circumjacent parts; and the muscular sphincter of its neck yielded readily to the introduction of the finger from the bladder into the urethra.

Uterus.—In its cavity was contained about a drachm of thick, darkish, fœtid pus, but no other appearance of disease in its body.

Tubæ Fallopianæ were larger than common in virgins, and strung with several hydatids or vesiculæ, the size of a walnut, filled with a watery glutinous humor. Corpora fimbriata had a gangrenous appearance.

Ovaria were enlarged to the size of a small hen's egg, and contained a considerable quantity of a clear limpid fluid immediately under the first coat.

