# Corrigenda

**Published:** 1813-12

**Authors:** 


					?? Viq. d AzyV) p. 501 ? Vjccj. d'/izyr*
?? Ditto, p. 502 ? Duio.

				

